# The association between weekly mean temperature and the epidemic of influenza across 122 countries/regions, 2014–2019

**DOI:** 10.7555/JBR.39.20250010

**Published:** 2025-04-25

**Authors:** Xiaoxiao Cao, Wenhao Zhu, Zhenghan Luo, Ran He, Yihao Li, Shirong Hui, Sheng Yang, Rongbin Yu, Peng Huang

**Affiliations:** 1 Department of Epidemiology, National Vaccine Innovation Platform, Center for Global Health, School of Public Health, Nanjing Medical University, Nanjing, Jiangsu 211166, China; 2 Department of Infectious Disease Prevention and Control Ⅰ, Center for Disease Control and Prevention, Eastern Theater of Operations, Nanjing, Jiangsu 210002, China; 3 Department of Biostatistics, National Vaccine Innovation Platform, Center for Global Health, School of Public Health, Nanjing Medical University, Nanjing, Jiangsu 211166, China

**Keywords:** influenza, influenza subtype, temperature, climatic zones, influenza transmission zones

## Abstract

The study examined the association between weekly mean temperature and influenza cases across 122 countries/regions (2014–2019) using a distributed lag non-linear model. We analyzed 3145206 cases of overall influenza (Flu-All), with influenza A (Flu-A) and influenza B (Flu-B) accounting for 73.49% and 26.51%, respectively. Within a lag of two weeks, Flu-All incidence demonstrated a bimodal temperature relationship, with peak relative risks (RR) of 6.02 (95% confidence interval [CI]: 1.92–20.77) at –8 ℃ and 3.08 (95% CI: 1.27–7.49) at 22 ℃. Flu-A exhibited a similar bimodal pattern, with RRs of 3.76 (95% CI: 2.39–5.91) at −8 ℃ and 2.08 (95% CI: 1.55–2.80) at 22 ℃. Flu-B demonstrated a single risk peak at 1 ℃ (RR = 4.48, 95% CI: 1.74–11.55). Subgroup analyses of climate zones revealed variations: tropical zones peaked at 12 ℃ (RR = 1.37, 95% CI: 1.08–1.74), while dry and temperate zones exhibited the highest risks at −5 ℃, with RRs of 4.49 (95% CI: 2.46–7.15) and 5.23 (95% CI: 3.17–8.64), respectively. Cold zones peaked at 1 ℃ (RR = 5.96, 95% CI: 3.76–9.43). Subgroup analyses of influenza transmission zones revealed variations: Africa showed a higher risk between 6 ℃ and 14 ℃, Asia showed a higher risk below 3 ℃, and Europe exhibited distinct risks of influenza peaks at −1 ℃ (Eastern Europe), 1 ℃ (Southwestern Europe), and −20 ℃ (Northern Europe). Elevated risks above 11 ℃ were identified in the Americas and Oceania. These findings establish a predictive framework for influenza outbreak preparedness by integrating regional temperature patterns with global climate variability.

## Introduction

Viral respiratory infections constitute a major global health burden. Alongside the recent COVID-19 pandemic^[1–2]^, seasonal influenza and COVID-19 represent significant respiratory diseases influenced by temperature. The World Health Organization (WHO) estimates that influenza causes 3–5 million severe cases annually, with 290000 to 650000 seasonal influenza-associated respiratory deaths worldwide^[3]^, presenting considerable health and economic challenges to society^[4]^.

In the context of global warming, rising temperatures significantly affect current and future respiratory diseases^[5]^. For example, studies in Australia have found an increasing burden of respiratory diseases caused by heat exposure in Perth^[6]^. Climate change has influenced viral vectors and the immune response of hosts (*e.g.*, young children and the elderly), altering the incidence and severity of respiratory infections^[7]^. Influenza, a major respiratory infection, is primarily caused by two main subtypes of viruses: influenza A (Flu-A) and influenza B (Flu-B), which are transmitted through direct or indirect aerosol/droplets^[8]^. Seasonality is a major feature in the spread of influenza, driven by various factors: weather influences the survival, activity, and transmission of the influenza virus^[9]^; uncomfortable ambient temperatures and rainfall often lead people to stay indoors, increasing indoor virus-host interactions and opportunities for influenza infections^[10]^; seasonal variations in host immunity may be affected by meteorological factors such as temperature and humidity through multiple processes^[11]^.

Temperature and precipitation, as key environmental determinants, significantly influence influenza virus transmission across climatic zones^[12]^. Using WHO's FluNet data, Muscatello *et al*^[13]^ characterized the seasonal variation of Flu-A and Flu-B in tropical and temperate zones between 2011 and 2017. Similarly, Newman *et al*^[14]^ analyzed six-year influenza patterns across 118 countries/regions, identifying bimodal epidemic peaks (summer/winter) through weekly FluNet surveillance data. Most studies have been conducted on small regional or national scales, with fewer examining the risk of influenza at the multinational or global level. The current study systematically evaluates the temperature-influenza associations at both regional and global levels.

## Materials and methods

### Data collection

Global influenza surveillance data were obtained from the WHO FluNet (https://www.who.int/tools/flunet)^[15]^, a standardized virological surveillance system that aggregates weekly laboratory-confirmed cases from National Influenza Centers worldwide. We downloaded and extracted information on the weekly reported number of influenza cases and the total number of samples tested per week for countries/regions between 2014 and 2019. The daily average temperature (℃) and precipitation (mm) for the 122 retained countries/regions were downloaded from the Global Historical Climatology Network daily (GHCNd, https://www.ncei.noaa.gov/products/land-based-station/global-historical-climatology-network-daily)^[16]^. Multi-station countries/regions underwent spatial averaging to create national-level estimates, which were temporally aggregated to a weekly resolution following ISO 8601 standards. We additionally acquired three socioeconomic covariates from Our World in Data^[17]^: (1) Human Development Index (HDI; quantifying health, education, and living standards), (2) gross domestic product (GDP), and (3) population density.

Data curation followed three sequential steps: (1) selection of complete influenza surveillance timelines (annual cumulative monitoring weeks ≥ 52); (2) exclusion of countries/regions with > 15% missing case data; and (3) retention of meteorological records with < 15% missingness. Missing climate data were imputed using multi-year, weekly means for corresponding country-week pairs.

### Study area

In the current study, based on the WHO global surveillance website FluNet, we identified 122 countries/regions that reported sufficient information on influenza to be suitable for time-series correlation model fitting. These countries/regions were stratified based on climate zones and influenza transmission patterns. First, based on the five principal Köppen climate classifications (A: tropical, B: dry, C: temperate, D: cold, and E: polar)^[18]^, they were divided into 12 subregions (A, AB, ABC, AC, B, BC, BCD, BDE, C, CD, D, and E), defined by the combination of more than 30% of the land area within the Köppen climate zone^[19]^. Furthermore, according to the document released by the WHO, these countries/regions were reclassified into 18 influenza transmission zones (ITZs)^[20]^, *i.e.*, Eastern Africa, Southern Africa, Western Africa, Northern Africa, Middle Africa, Eastern Asia, Southern Asia, Western Asia, Central Asia, Southeast Asia, Eastern Europe, Southwestern Europe, Northern Europe, temperate South America, tropical South America, North America, Central America and the Caribbean, and Oceania Melanesia and Polynesia. Detailed listings of the countries/regions within each subzone are provided in ***Supplementary Table 1*** (available online).

### Statistical analysis

We examined the association between temperature and influenza through a two-stage analysis approach^[21]^. In the first stage, we fitted a quasi-Poisson generalized linear model for each country as follows^[22]^:

\begin{equation*}\begin{split}
 & \mathrm{lg}[\mathrm{E}\left(Y_t\right)]=a+\mathrm{cb}\left(Temp,lag\right)+\mathrm{ns}\left(time,df\right)+ \\ &\mathrm{MOY}_t+\mathrm{Precipitation}+\mathrm{Population}_{\mathrm{density}}+ \\ &\mathrm{HDI}+\mathrm{GDP}+\mathrm{Speccessed}_{\mathrm{NB}}.
\end{split}\end{equation*}

The model equation estimated cumulative relative risk ratios (RR) and 95% confidence intervals (95% CIs) between temperature and influenza cases, adjusting for variables such as time, each country's population, and development, with 10 ℃ for the weekly average temperature set as the reference.

Where E(*Yt*) indicates the number of influenza cases reported in week t, cb(*Temp*, *lag*) is the cross-basis matrix of weekly mean temperature and the lagged effect. The cross-basis term is constructed to assess the association between temperature and influenza under the distributed lag nonlinear model, which consists of a quadratic B-spline with three internal knots placed at the 25^th^, 50^th^, and 75^th^ percentiles of country/region-specific temperature distributions for the exposure-response dimension and a natural spline with one equally spaced internal knot in the log scale of the lag dimension^[23]^. The maximum lag is three weeks, accounting for the incubation period and duration of influenza^[24]^. ns(*time*, *df*) denotes a natural spline function to control for long-term trends and seasonal variations; *df* denotes the degrees of freedom. MOY_*t*_ is an indicator variable for the month of the year, and covariates include precipitation, population density, HDI per capita, GDP per capita, and Specessed_NB (the total number of influenza samples tested in week t). The models above estimated the cumulative RR and 95% CI between temperature and influenza cases.

In the second stage, we pooled RRs between temperature and influenza at the global and subregional levels using mixed-effects meta-analyses, which allow more complex random effects that may account for variation in risk at the level of two nested subgroups^[25]^. The Best Linear Unbiased Predictors (BLUPs) borrow information from the entire sample to produce more accurate cumulative RR estimates, especially for those with higher uncertainty, while accounting for heterogeneity in risks^[26]^. Unlike fixed effects, which assign the same weight to each effect, random effects using the inverse variance method give more weight to smaller studies when there is heterogeneity, resulting in wider CIs for meta-analyses of the mean effect and a greater preference for the inclusion of subjects that represent the entire population^[27]^. Thus, we also obtained the BLUP for each country to assess the consistency of cumulative RR between temperature and influenza cases in each country at the pooled sub-regional and global levels.

Subsequently, we adopted the temperatures corresponding to the lowest influenza risk from the cumulative risk curves previously constructed, using 10 ℃ as the reference temperature, and calculated the cumulative risk curves for different climate zones and influenza transmission regions.

### Sensitivity analysis

To stabilize control of long-term trends, we selected degrees of freedom ranging from one to eight, determining the optimal degree based on the Akaike information criterion (AIC) value. We analyzed the cumulative RRs between temperature and influenza cases at lag times of 0, 1, 2, and 3 weeks to identify the lag week corresponding to the maximum RRs at the global level. We further examined the relationship between temperature and influenza cases during the lag-week period of maximum RRs across climate zones and ITZs.

All data analyses were performed using R software, version 4.3.0 (R Project for Statistical Computing, Vienna, Austria, http://www.r-project.org). The distributed lag non-linear model and mixed-effects meta-analysis were fitted using the 'dlnm' and 'mixmeta' packages, respectively.

## Results

### Descriptive analysis

**Figure 1:**
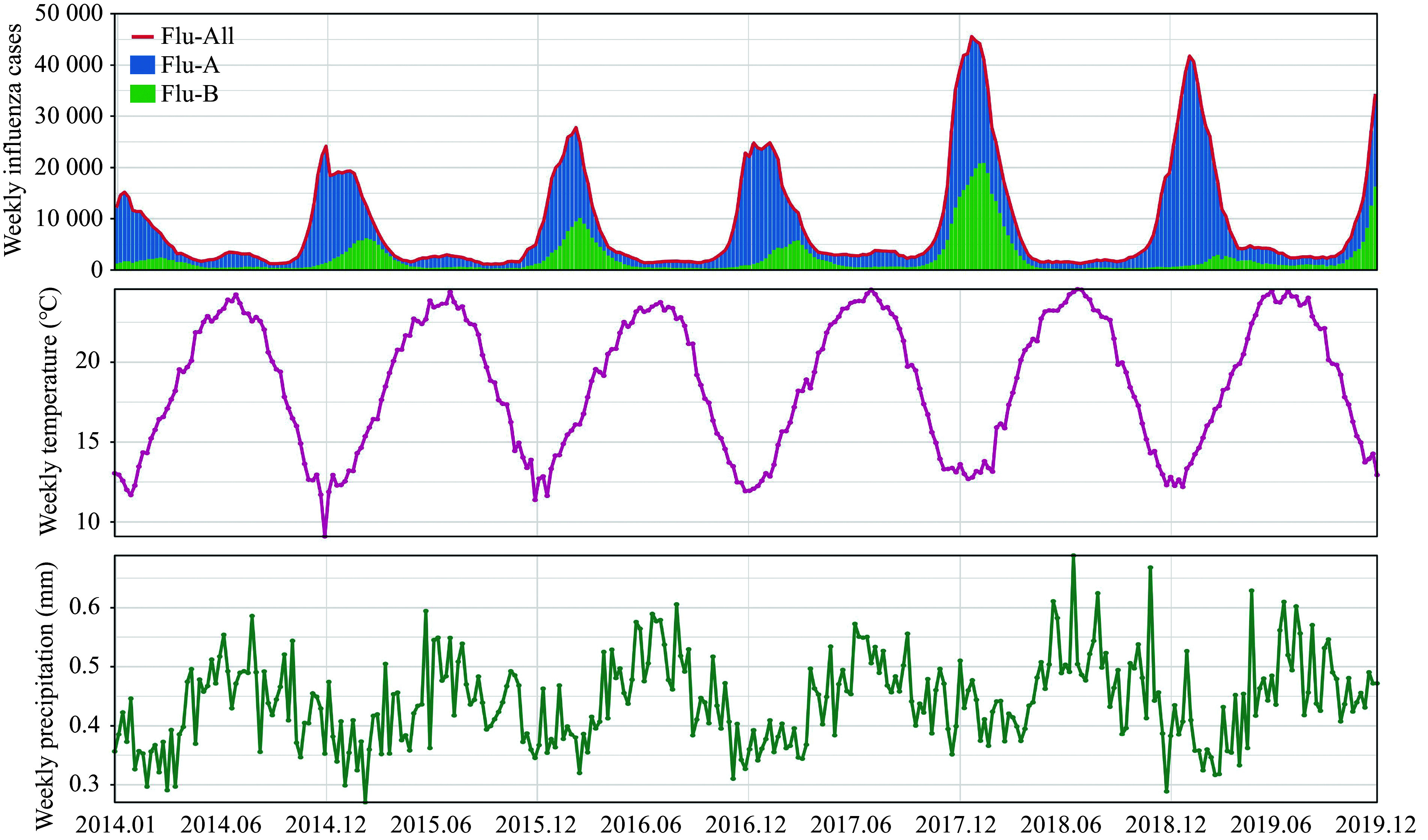
Time series of weekly Flu-All/Flu-A/Flu-B of 122 countries/regions between 2014 and 2019. Abbreviations: Flu-All, all virus types for influenza A and influenza B; Flu-A, influenza A; Flu-B, influenza B.

This study analyzed a total of 3145206 influenza cases (Flu-All), including 2311446 (73.49%) cases of influenza A (Flu-A) and 833760 (26.51%) cases of influenza B (Flu-B) across 122 countries/regions. ***Supplementary Table 2*** (available online) summarizes the weekly cases of influenza in these countries/regions between 2014 and 2019. The mean (± standard deviation) number of weekly Flu-All cases was 93.05 (± 658.03). The weekly mean temperature across 122 countries/regions ranged from a minimum of –29.16 ℃ to a maximum of 41.63 ℃, with a mean and standard deviation of 18.15 ℃ and 10.41 ℃, respectively. The mean and standard deviation of the weekly mean precipitation for these countries/regions were 0.41 mm and 0.58 mm, respectively. The United States reported the highest number of influenza cases, with 1036844 cases, followed by mainland China, with 402739 cases. Time series analyses revealed a clear seasonal pattern in the weekly influenza cases, with Flu-A predominating in the influenza epidemic (***Fig. 1***).

### Overall cumulative RRs of influenza cases with respect to temperature and lag at the global level

The cumulative RR curves between temperature and influenza cases (Flu-All, Flu-A, and Flu-B) showed nonlinear relationships at different lags (0–1, 0–2, and 0–3 weeks), with cumulative RRs being higher in the low-temperature range than in the high-temperature range. Flu-A exhibited a higher peak RR than Flu-B, and its second peak occurred at a lower temperature compared with Flu-B (***Supplementary Fig. 1***, available online). The cumulative risk of temperature and influenza was higher at a lag of 0–2 weeks than at other lag times, with a narrower CI. Flu-A peaked at lower temperatures than Flu-B. The cumulative RR curve within a 0–2 week lag period demonstrated that RR values exhibited statistically significant associations across a broader temperature range (***Table 1***). Therefore, we selected a lag time of 2 weeks as the most optimal.

**Table 1:** The highest and lowest RRs for influenza and the corresponding temperature at the global level

Total region	Lag weeks	RT (℃)	ST (℃)	Peak 1			Peak 2	
				Temperature (℃)	RR (95% CI)		Temperature (℃)	RR (95% CI)
Flu-All	0	35	−29–31	−10	4.81 (2.67, 7.27)^*^		–	–
	1	35	−26–32	−9	6.51 (2.63, 16.12)^*^		–	–
	2	36	−19–31	−8	6.23 (1.92, 20.77)^*^		22	3.08 (1.27, 7.49)^*^
	3	39	−20–27	−8	6.77 (2.38, 19.27)^*^		22	3.06 (1.93, 4.86)^*^
Flu-A	0	35	−29–31	−15	3.96 (2.64, 5.94)		–	–
	1	35	−28–32	−10	4.70 (2.97, 7.44)^*^		–	–
	2	35	−21–28	−8	3.76 (2.39, 5.91)^*^		22	2.08 (1.55, 2.80)^*^
	3	35	−17–11, 21–27	−7	3.45 (2.03, 5.87)^*^		–	–
Flu-B	0	36	−29–32	12	3.52 (2.50, 4.96)^*^		–	–
	1	36	−29–31	3	4.67 (2.40, 9.07)^*^		–	–
	2	35	−29–31	1	4.48 (1.74, 11.55)^*^		–	–
	3	36	−29–29	0	4.34 (1.66, 11.37)^*^		–	–
Sensitive temperature (ST) indicates a statistically significant temperature range. En dash (–) indicates the absence of peak risk. ^*^Statistically significant with a 95% CI range > 1. Abbreviations: RT, reference temperature; Flu-All, all virus types for influenza A and influenza B; Flu-A, influenza A; Flu-B, influenza B; RR, relative risk; CI, confidence interval.								

**Figure 2:**
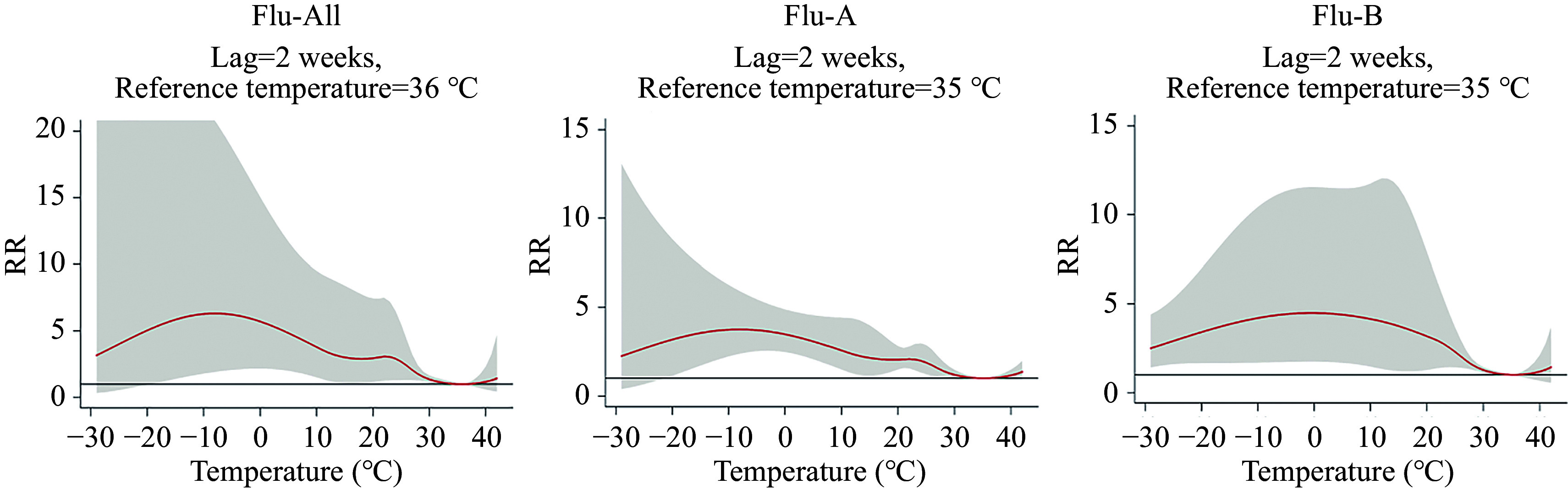
Overall cumulative RR curves and 95% CI between Flu-All, Flu-A, Flu-B, and temperature at the global level (lag 2 weeks). The red solid line represents the cumulative relative risk (RR), shaded for the 95% confidence interval (CI). Abbreviations: Flu-All, all virus types for influenza A and influenza B; Flu-A, influenza A; Flu-B, influenza B.

At a lag of 2 weeks, the cumulative RR curves between temperature and influenza cases demonstrated distinct patterns (***Fig. 2***): an M-shaped relationship for Flu-All and Flu-A, with sensitive temperature ranges of –19° to 31 ℃ for Flu-All and –21 ℃ to 28 ℃ for Flu-A, and an inverted U-shaped relationship for Flu-B, with the sensitive temperature range of –29 ℃ to 31 ℃. Both Flu-All and Flu-A exhibited two peaks at –8 ℃ and 22 ℃, whereas Flu-B peaked at 1 ℃. The cumulative RRs increased steadily up to the first peak of –8 ℃, with RR = 6.23 (95% CI: 1.92, 20.77) for Flu-All, and RR = 3.76 (95% CI: 2.39, 5.91) for Flu-A; then decreased before increasing again to the second peak at 22 ℃, with RR = 3.06 (95% CI: 1.93, 4.86) for Flu-All, and RR = 2.08 (95% CI: 1.55, 2.80) for Flu-A. For Flu-B, the maximum RR was observed at 1 ℃, with RR = 4.48 (95% CI: 1.74, 11.55). The reference temperatures were approximately 36 ℃ for Flu-All, and around 35 ℃ for Flu-A and Flu-B, as determined by BLUPs (***Supplementary Fig. 2***, available online).

### Overall cumulative RRs of influenza cases to temperature in different regions

We further investigated the differences in cumulative RRs between influenza cases and temperature across various climatic zones and ITZs. The observed practical temperature ranges showed significant differences, leading to different sensitive temperature thresholds across regions (***Table 2*** and ***Table 3***). The global temperature range (–30 ℃ to 42 ℃) encompassed the practical temperatures observed in all regions, so we used this range to uniformly predict the cumulative RR curves summarized for each region.

**Table 2:** Description of temperature and peak risk of influenza in climatic zones (lag 2 weeks)

Zones	PT (℃)	Flu-All risk peak					Flu-A risk peak					Flu-B risk peak			
		RT (℃)	ST (℃)	T (℃)	RR (95% CI)		RT (℃)	ST (℃)	T (℃)	RR (95% CI)		RT (℃)	ST (℃)	T (℃)	RR (95% CI)
A	11–34	34	−10–22	12	1.37 (1.08, 1.74)^*^		33	−29–28	11	1.40 (1.03, 1.90)^*^		34	9–21	16	1.41 (1.11, 1.80)^*^
AB	12–35	35	−29–13	12	1.87 (1.03, 3.40)^*^		35	−29–10	12	2.20 (0.95, 5.10)		35	−25–−15	15	1.50 (0.69, 3.26)
ABC	−7–33	33	–	23	2.25 (0.47, 10.72)		28	–	7	1.48 (0.81, 2.71)		33	–	24	1.49 (0.59, 3.78)
AC	16–34	34	–	24	1.68 (0.78, 3.58)		16	–	−3	1.43 (0.88, 2.30)		16	–	25	1.22 (0.63, 2.35)
B	−29–42	36	−29–26	−5	4.19 (2.46, 7.15)^*^		39	−26–21	−5	4.04 (2.15, 7.60)^*^		34	−23–29	10	3.65 (2.31, 5.75)^*^
BC	6–29	28	−10–22	12	8.58 (2.99, 24.66)^*^		29	−27–22	12	10.92 (3.82, 31.18)^*^		29	−10–21	13	4.37 (2.09, 9.12)^*^
BCD	−7–33	33	−29–21	12	6.97 (2.41, 20.20)^*^		26	−29–22	−7	7.32 (2.19, 24.48)^*^		33	−13–20	5	3.99 (1.95, 8.15)^*^
BDE	−13–26	26	−9–20	12	28.62 (5.70, 143.84)^*^		26	−7–−6, 20−21	−7	22.62 (2.03, 252.13)^*^		26	6–20	6	–
C	−5–30	30	−29–30	−5	5.23 (3.17, 8.64)^*^		30	−29–30	−5	8.62 (4.70, 15.77)^*^		30	−29–24	14	3.14 (1.63, 6.05)^*^
CD	−9–28	28	−29–28	5	5.70 (2.84, 11.43)^*^		28	−29–28	−15	7.72 (2.76, 21.60)^*^		28	−10–23	13	5.93 (2.16, 16.23)^*^
D	−24–30	30	−29–30	1	5.96 (3.76, 9.43)^*^		30	−29–30	−6	10.47 (6.06, 18.12)^*^		30	−21–30	22	3.92 (2.63, 5.85)^*^
E	−5–13	13	–	−5	1.16 (0.64, 2.13)		13	–	−5	1.21 (0.62, 2.36)		−5	–	15	1.65 (0.31, 8.77)
A, B, C, D, and E indicate tropical, dry, temperate, cold, and polar climates, respectively. Sensitive temperature (ST) indicates a statistically significant temperature range. En dash (–) indicates data that is not present or not epidemiologically significant.^*^Statistically significant with a 95% CI range >1.Abbreviations: PT, practical temperature range; RT, reference temperature; T, peak temperature; Flu-All, all virus types for influenza A and influenza B; Flu-A, influenza A; Flu-B, influenza B; RR, relative risk; CI, confidence interval.															

**Table 3:** Description of temperature and peak risk of influenza in influenza transmission zones (lag 2 weeks)

Zones	PT (℃)	Flu-All risk peak					Flu-A risk peak					Flu-B risk peak			
		RT (℃)	ST (℃)	T (℃)	RR (95% CI)		RT (℃)	ST (℃)	T (℃)	RR (95% CI)		RT (℃)	ST (℃)	T (℃)	RR (95% CI)
Eastern Africa	19–29	19	–	29	1.22 (0.72, 2.07)		19	–	29	1.36 (0.73, 2.54)		30	–	23	1.54 (0.74, 3.20)
Southern Africa	7–24	24	−9–24	13	2.68 (1.55, 4.65)^*^		24	17–24	14	2.87 (0.93, 8.89)		24	–	7	2.03 (0.96, 4.27)
Western Africa	16–36	36	2–21	14	1.93 (1.24, 2.99)^*^		36	–	14	2.42 (0.67, 8.73)		36	−1–32	21	5.71 (1.66, 19.65)^*^
Northern Africa	7–34	34	−18–25	6	9.42 (5.48, 16.22)^*^		33	−18–22	0	12.85 (6.27, 26.36)^*^		34	−6–23	12	4.28 (2.07, 8.82)^*^
Middle Africa	20–33	25	–	20	1.71 (0.97, 3.01)		33	–	1	6.63 (0.19, 229.84)		25	–	14	2.82 (0.66, 12.04)
Eastern Asia	−29–30	30	−29–21	−9	5.80 (2.27, 14.85)^*^		30	−29–17	7	5.30 (1.75, 16.07)^*^		30	−29–30	13	4.61 (2.29, 9.31)^*^
Southern Asia	−2–34	34	–	−2	1.60 (0.47, 5.46)		24	–	−2	1.49 (0.44, 5.10)		34	–	1	1.84 (0.64, 5.32)
Western Asia	−13–42	39	−29–28	3	6.31 (3.17, 12.58)^*^		36	−29–27	−13	8.87 (3.09, 25.48)^*^		36	−21–29	14	4.52 (2.43, 8.38)^*^
Central Asia	−21–27	25	−19–20	−19	92.69 (1.08, 67.51)^*^		27	−15–0	−15	15.10 (1.15, 198.67)^*^		27	−10–17	4	25.47 (6.01, 107.99)^*^
Southeast Asia	17–34	34	–	22	1.26 (0.86, 1.87)		32	–	23	1.48 (0.81, 2.71)		34	–	3	1.20 (0.74, 1.99)
Eastern Europe	−24–26	26	−14–26	−1	2.25 (1.29, 3.93)^*^		26	−21–26	−4	4.57 (2.16, 9.65)^*^		−24	13–26	23	4.32 (1.67, 11.18)^*^
Southwestern Europe	−9–30	30	−29–30	11	5.80 (3.87, 8.70)^*^		30	−29–30	−8	7.15 (4.23, 12.08)^*^		30	−12–30	14	3.84 (1.80, 8.17)^*^
Northern Europe	−20–24	24	−29–24	−20	3.51 (1.78, 6.92)^*^		24	−29–24	−20	5.83 (3.27, 10.39)^*^		24	22–24	−10	1.67 (0.70, 3.99)
Temperate South America	7–31	31	−25–22	13	3.80 (2.73, 5.29)^*^		31	−29–24	13	7.04 (4.20, 11.77)^*^		25	−5–25	11	3.27 (1.83, 5.84)^*^
Tropical South America	17–31	31	−29–19	17	1.43 (1.11, 1.84)^*^		31	−29–21	17	1.76 (1.19, 2.61)^*^		24	–	17	1.71 (0.71, 1.93)
North America	−21–21	21	−29–21	11	2.24 (1.75, 2.87)^*^		21	−29–21	−21	3.91 (2.74, 5.60)^*^		−21	−21–14	4	1.90 (1.12, 3.18)^*^
Central America Caribbean	16–32	32	−29–28	16	2.44 (1.04, 5.74)^*^		32	−29–13, 16–32	16	2.74 (1.01, 7.48)^*^		32	12–22	19	2.10 (1.29, 3.41)^*^
Oceania Melanesia and Polynesia	6–30	30	−29–8, 18–22	6	1.45 (1.10, 1.91)^*^		30	–	24	1.90 (0.71, 5.10)		30	−29–−9, 12–21	16	2.48 (1.50, 4.09)^*^
				22	1.71 (1.00, 2.93)^*^										
Sensitive temperature (ST) indicates a statistically significant temperature range. En dash (–) inidcates data that is not present or not epidemiologically significant.^*^Statistically significant with a 95% CI range > 1.Abbreviations: PT, practical temperature range; RT, reference temperature; T, peak temperature; Flu-All, all virus types for influenza A and influenza B; Flu-A, influenza A; Flu-B, influenza B; RR, relative risk; CI, confidence interval.															

#### Köppen climate zones

We divided the 122 countries/regions into 12 subregions based on the five main types of Köppen climate zones to assess cumulative RR curves between temperature and influenza cases in each region (***Table 2***). For Flu-All, nine regions showed statistically significant differences within the practical temperature range. We found that zones A, AB, BC, BCD, and BDE had peak RRs at 12 ℃, with RRs (95% CI) of 1.37 (1.08, 1.74), 1.87 (1.03, 3.40), 8.58 (2.99, 24.66), 6.97 (2.41, 20.20), and 28.62 (5.70, 143.84), respectively. Zones B and C had peak RRs at –5 ℃, with RRs (95% CI) of 4.19 (2.46, 7.15) and 5.23 (3.17, 8.64), respectively. The RR (95% CI) corresponding to the peak at 5 ℃ in zone CD was 5.70 (2.84, 11.43), and the peak at 1 ℃ in zone D had an RR (95% CI) of 5.96 (3.76, 9.43). For Flu-A, the results showed statistically significant differences in eight regions, whereas Flu-B exhibited statistically significant differences in only seven regions. Each exhibited a single peak risk; the temperatures corresponding to the peak RRs in each region of Flu-A were lower than those for Flu-B. Notably, Flu-A and Flu-B showed different peak temperatures for influenza risk across these climate zones, whereas Flu-All showed two distinct temperature peaks for overall influenza risk (***Fig. 2***), indicating a combined effect from both subtypes.

#### ITZs

The ITZs refer to geographical groups of countries/regions, areas, or territories with similar influenza transmission patterns. Within the practical temperature range, the cumulative RR curves between temperature and influenza cases in the 18 regions generally followed an inverted U-shape. However, the temperatures corresponding to the peak RR varied by region (***Table 3***).

For Flu-All, a total of 14 regions exhibited statistically significant associations between temperature and influenza cases. The temperatures and corresponding RRs (95% CIs) at which maximum risk occurred in each region were as follows: Southern Africa peaked at 13 ℃ with an RR of 2.68 (1.55, 4.65), Western Africa peaked at 14 ℃ with an RR of 1.93 (1.24, 2.99), Northern Africa peaked at 6 ℃ with an RR of 9.42 (5.48, 16.22), Eastern Asia peaked at –9 ℃ with an RR of 5.80 (2.27, 14.85), Western Asia peaked at 3 ℃ with an RR of 6.31 (3.17, 12.58), Central Asia peaked at −19 ℃ with an RR of 92.69 (1.08, 67.51), Eastern Europe peaked at –1 ℃ with an RR of 2.25 (1.29, 3.93), Southwestern Europe peaked at 11 ℃ with an RR of 5.80 (3.87, 8.70), Northern Europe peaked at –20 ℃ with an RR of 3.51 (1.78, 6.92), temperate South America peaked at 13 ℃ with an RR of 3.80 (2.73, 5.29), tropical South America peaked at 17 ℃ with an RR of 1.43 (1.11, 1.84), North America peaked at 11 ℃ with an RR of 2.24 (1.75, 2.87), Central America Caribbean peaked at 16 ℃ with an RR of 2.44 (1.04, 5.74), and Oceania Melanesia and Polynesia had two peaks at 6 ℃ with an RR of 1.45 (1.10, 1.91) and 22 ℃ with an RR of 1.71 (1.00, 2.93). For Flu-A, cumulative RR curves exhibited patterns similar to those of Flu-All; however, Southern Africa, Western Africa, and Oceania Melanesia and Polynesia did not show statistically significant associations. For Flu-B, the peak RRs generally occurred at higher temperatures than those for Flu-A. Additionally, no statistically significant differences were observed for Flu-B in Eastern, Southern, and Middle Africa; Southern and Southeast Asia; Northern Europe; and tropical South America.

## Discussion

Global temperature changes remind us to observe the shifting trends in influenza risk peaks from a global perspective, providing theoretical support for timely predictions of influenza outbreaks both globally and in specific regions. Our results align with those of Dai *et al*^[28]^ regarding the impact of influenza activity in Jiangsu Province of southern China between 2013 and 2016, where Flu-A had two peaks at −2 ℃ and 28 ℃ based on a reference temperature of 16 ℃, while Flu-B had a peak at 5 ℃ with temperatures ranging from −8.03 ℃ to 33.48 ℃ based on a reference temperature of 22 ℃. The current study, which spans 122 countries/regions, takes into account local climate, geography, and demographic characteristics and highlights significant regional differences. For example, even within Australia, regions like Sydney (Eastern Australia) and Perth (Western Australia) exhibit different reference and peak risk temperatures^[6]^. The lowest global influenza risk was observed at temperatures of 35 ℃ and 36 ℃, which were used as reasonable reference temperatures for this study.

In a similar study conducted by Bai *et al*^[29]^ between 2012 and 2015 in Huludao, a city in northern China, influenza-like illness peaked at −13 ℃ and 18 ℃. This double peak relationship between temperature and influenza epidemics has been demonstrated in several temperate and subtropical regions, including Shanghai (China)^[30]^ and Okinawa (Japan)^[31]^. However, not all regions exhibit this pattern. For example, a study in Hangzhou (Zhejiang province, China) between 2014 and 2018 showed that peak influenza risk occurred at 30.5 ℃, despite temperatures ranging from −0.8 ℃ to 33.74 ℃^[32]^. Studies in British Columbia also found a peak influenza risk at 5 ℃, with temperatures ranging from −5 ℃ to 25 ℃^[30]^. Additionally, a study across 48 U.S. states conducted by Yang and colleagues^[33]^ observed that within a weekly temperature range of −15 ℃ to 35 ℃, −2.59 ℃ triggered a higher risk of influenza. These studies demonstrate that influenza epidemics are influenced by climatic zones and influenza subtypes^[34]^, aligning with our findings that regional temperatures play a significant role in shaping epidemic patterns. We further determined the association between weekly temperatures and influenza pandemic risk in different climatic zones.

The temperature ranges within Köppen climate zones are not uniform, but several zones experienced similar temperatures at the peak of the influenza epidemic. Differences in regional temperature ranges are associated with the extent of solar radiation exposure at different latitudes, leading to distinct climatic zones. Our findings showed the peak influenza epidemic temperature at 12 ℃ in the A, AB, BC, BCD, and BDE zones, −5 ℃ in the B, C, and E zones, and 5 ℃ in the CD zone. These results align with those of Lowen *et al*^[35]^, which suggest that influenza virus aerosol transmission efficiency decreases as the temperature rises from 5 ℃ to 20 ℃, ceasing completely at 30 ℃. Despite regional variation in temperature range, the peak temperatures corresponding to influenza risk consistently ranged from −8 ℃ to 22 ℃ across the 122 countries/regions. Our findings are in agreement with previous studies on influenza risk and temperature^[36]^, supporting the conclusion that influenza risk is influenced by both climatic factors and influenza subtypes.

We also observed that areas with a single climatic feature had narrower CIs for peak influenza pandemic risk. This may be because the prevalence of influenza viruses is influenced not only by climate zones but also by other factors, such as the hygiene habits of the inhabitants at different altitudes, which in turn creates a specific pattern of influenza transmission. To further assess these patterns, we examined the association between influenza risk and temperature in different ITZs.

Our analysis revealed that the ITZ exhibited narrower CIs for peak influenza epidemic risk compared with Köppen climate zones. Tropical and subtropical regions of East and Central Africa, Eastern Europe, and Oceania displayed two RR peaks, while regions like North and South Africa, temperate East and West Asia, Southwestern and Northern Europe, and Central and South America showed only one peak. These findings are consistent with studies by Chong *et al*^[34]^, which underscore the pivotal role of geographic and climatic factors in shaping influenza transmission dynamics.

Despite the valuable insights from the current study, several limitations exist. First, the dynamics of influenza pandemics are influenced by multiple factors, including air pollution^[37]^ and public health infrastructure^[38]^, which could explain part of the observed increase in risk. Second, we did not consider factors like host susceptibility and seasonal vaccination in influenza incidence^[9]^. Furthermore, the quality of influenza surveillance data may vary because of differences in sentinel site protocols and patient behavior^[39]^, which may affect the reliability of the data. The lack of data on air conditioning also limits our ability to account for its impact on influenza transmission, which may vary across different regions. These factors will be explored in more depth in future studies.

In conclusion, the current study evaluated the exposure-lag response relationship between temperature and influenza cases in 122 countries/regions using a two-stage analytic procedure. We found that Flu-A dominated influenza activity globally, with peaks occurring at both low (−8 ℃) and high (22 ℃) temperatures under an exposure lag of 0–2 weeks. Flu-B, on the other hand, had a single peak at low temperatures (1 ℃), with higher temperature peaks observed for Flu-A. The temperatures corresponding to peak influenza activity within climate zones and ITZs were influenced by the practical temperature range, and the pandemic patterns in the ITZs provided a more realistic representation of influenza transmission. The study's results may help predict influenza outbreaks, issue early warnings, and develop prevention strategies to reduce the risk of influenza associated with global and regional temperature changes.

## SUPPLEMENTARY DATA

Supplementary data to this article can be found online.
