# Supplementary material for: The association between weekly mean temperature and the epidemic of influenza across 122 countries/regions, 2014–2019
Source: J Biomed Res. 2025 Apr 25;39(6):601–10. doi: 10.7555/JBR.39.20250010 (PMC12683506; doi:10.7555/JBR.39.20250010)
Supplement: Supplementary file 1 — Supplementary data to this article can be found online. [file jbr-39-6-601-Supplementary.pdf]

Available online at www.jbr-pub.org.cn

Open Access at PubMed Central

JBR

Journal of Biomedical Research, 2025 39(6): 1–2

Supplementary Data

The association between weekly mean temperature and the epidemic of influenza across 122 countries/regions, 2014–2019

Xiaoxiao Cao[1,△], Wenhao Zhu[1,△], Zhenghan Luo[2], Ran He[1], Yihao Li[1], Shirong Hui[1], Sheng Yang[3], Rongbin Yu[1,✉], Peng Huang[1,✉]

[1]Department of Epidemiology, National Vaccine Innovation Platform, Center for Global Health, School of Public Health, Nanjing Medical University, Nanjing, Jiangsu 211166, China;
[2]Department of Infectious Disease Prevention and Control Ⅰ, Center for Disease Control and Prevention, Eastern Theater of Operations, Nanjing, Jiangsu 210002, China;
[3]Department of Biostatistics, National Vaccine Innovation Platform, Center for Global Health, School of Public Health, Nanjing Medical University, Nanjing, Jiangsu 211166, China.

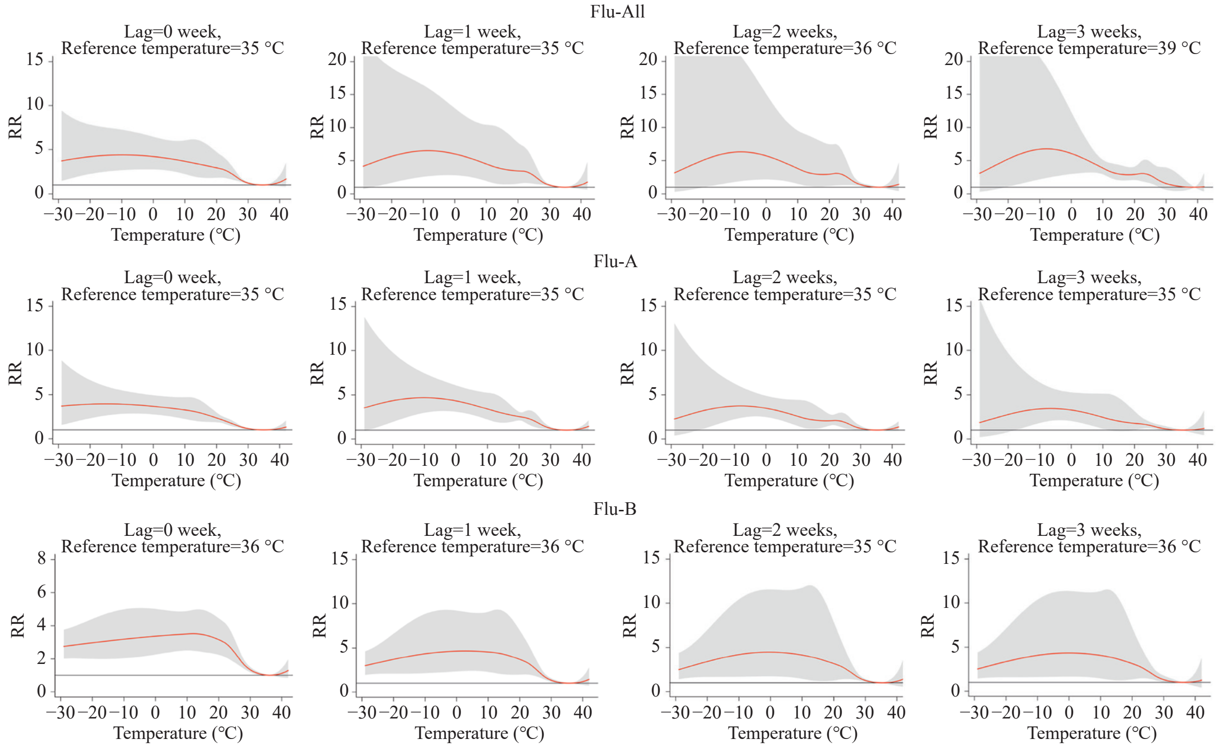

Supplementary Fig. 1 **Exposure-response risk plots of temperature versus influenza cases at the global level with lag times of 0, 1, 2, and 3 weeks.** Abbreviations: Flu-All, all virus types for influenza A and influenza B; Flu-A, influenza A; Flu-B, influenza B; RR, relative risk; shaded for 95% CI, confidence interval.

△These authors contributed equally to this work.

✉Corresponding authors: Rongbin Yu and Peng Huang, Department of Epidemiology, National Vaccine Innovation Platform, Center for Global Health, School of Public Health, Nanjing Medical University, Nanjing, Jiangsu 211166, China. E-mails: rongbinyu@njmu.edu.cn (Yu) and huangpeng@njmu.edu.cn (Huang).

Received: 05 January 2025; Revised: 08 April 2025; Accepted: 16 April 2025; Published online: 25 April 2025

CLC number: R511.7, Document code: A

The authors reported no conflict of interests.

This is an open access article under the Creative Commons Attribution (CC BY 4.0) license, which permits others to distribute, remix, adapt and build upon this work, for commercial use, provided the original work is properly cited.

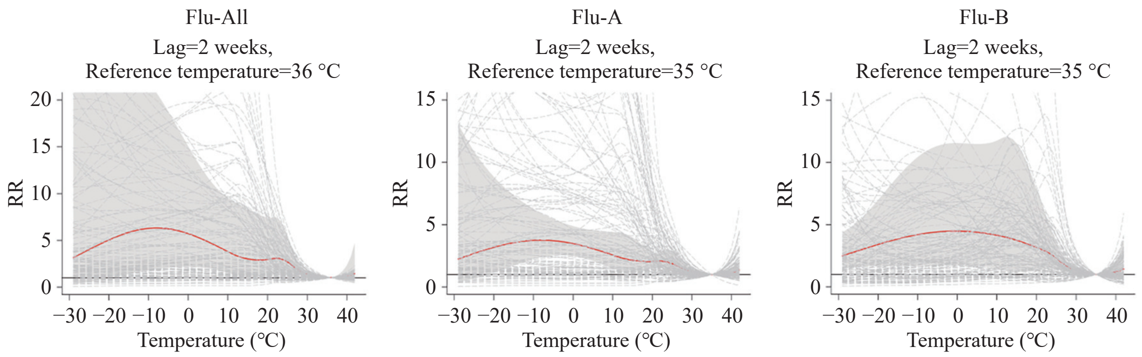

Supplementary Fig. 2 **Best linear unbiased predictors of exposure-response relationships for cumulative relative risk between temperature and influenza in 122 countries (lag 0–2 weeks).** Abbreviations: Flu-All, all virus types for influenza A and influenza B; Flu-A, influenza A; Flu-B, influenza B; RR, relative risk.

Supplementary Table 1 **Details of Köppen climate zone and influenza transmission zones for 122 countries/regions**

Zone	Countries/regions
Köppen climate zone[a]	
A (34)	Belize, Brazil, Cambodia, Cameroon, Central African Republic, Colombia, Costa Rica, Cuba, Côte d'Ivoire, Democratic Republic of the Congo, Dominican Republic, Egypt, El Salvador, Fiji, Ghana, Guinea, Honduras, Indonesia, Jamaica, Malaysia, Maldives, Morocco, Mozambique, Nicaragua, Nigeria, Papua New Guinea, Philippines, Singapore, Sri Lanka, Thailand, Togo, Trinidad and Tobago, United Republic of Tanzania, Venezuela
AB (4)	Bolivia, Burkina Faso, Kenya, Paraguay
ABC (5)	Ecuador, India, Mauritius, North Macedonia, Peru
AC (4)	Bangladesh, Lao People's Democratic Republic, Rwanda, Viet Nam
B (19)	Algeria, Australia, Bahrain, Iran, Iraq, Jordan, Kazakhstan, Kuwait, Mali, Mexico, Montenegro, Niger, Oman, Pakistan, Qatar, Saudi Arabia, Senegal, Tunisia, Uzbekistan
BC (5)	Argentina, Greece, Israel, South Africa, Spain
BCD (6)	Afghanistan, Azerbaijan, Chile, mainland China, Türkiye, United States of America
BDE (2)	Kyrgyzstan, Tajikistan
C (16)	Belgium, Cyprus, Denmark, France, Germany, Ireland, Italy, Lebanon, Luxembourg, Malta, Nepal, Netherlands, New Zealand, Portugal, United Kingdom, Uruguay
CD (9)	Bulgaria, Croatia, Georgia, Hungary, Japan, Mongolia, Serbia, Slovenia, Switzerland
D (17)	Armenia, Austria, Belarus, Canada, Czechia, Estonia, Finland, Latvia, Lithuania, Norway, Poland, Republic of Korea, Romania, Russian Federation, Slovakia, Sweden, Ukraine
E (1)	Iceland
Influenza transmission zones	
Eastern Africa (5)	Kenya, Mauritius, Mozambique, Rwanda, United Republic of Tanzania
Southern Africa (1)	South Africa
Western Africa (9)	Burkina Faso, Côte d'Ivoire, Ghana, Guinea, Mali, Niger, Nigeria, Senegal, Togo
Northern Africa (4)	Algeria, Egypt, Morocco, Tunisia
Middle Africa (3)	Cameroon, Central African Republic, Democratic Republic of the Congo
Eastern Asia (4)	Japan, mainland China, Mongolia
Southern Asia (8)	Afghanistan, Bangladesh, India, Iran, Maldives, Nepal, Pakistan, Sri Lanka
Western Asia (13)	Armenia, Azerbaijan, Bahrain, Cyprus, Georgia, Iraq, Israel, Jordan, Kuwait, Lebanon, Oman, Qatar, Saudi Arabia
Central Asia (4)	Kazakhstan, Kyrgyzstan, Tajikistan, Uzbekistan
South East Asia (8)	Cambodia, Indonesia, Lao People's Democratic Republic, Malaysia, Philippines, Singapore, Thailand, Viet Nam
Eastern Europe (8)	Belarus, Czechia, Hungary, Poland, Romania, Russian Federation, Slovakia, Ukraine
South West Europe (17)	Austria, Belgium, Croatia, France, Germany, Greece, Italy, Luxembourg, Malta, Montenegro, Netherlands, North Macedonia, Portugal, Serbia, Slovenia, Spain, Switzerland
Northern Europe (10)	Denmark, Estonia, Finland, Iceland, Ireland, Latvia, Lithuania, Norway, Sweden, United Kingdom
North America (2)	Canada, United States of America
Central America Caribbean (12)	Belize, Bulgaria, Costa Rica, Cuba, Dominican Republic, El Salvador, Honduras, Jamaica, Mexico, Nicaragua, Trinidad and Tobago, Türkiye
Temperate South America (4)	Argentina, Chile, Paraguay, Uruguay
Tropical South America (6)	Bolivia, Brazil, Colombia, Ecuador, Peru, Venezuela
Oceania Melanesia and Polynesia (4)	Australia, Fiji, New Zealand, Papua New Guinea

[a]A = Tropical, B = Dry, C = Temperate, D = Cold, E = Polar.

Supplementary Table 2 (available online) shows the descriptive statistics of weekly cases of influenza in different states across 122 countries/regions, 2014–2019.
